# Comparing Complication Rates for Plastic Surgery Minor Procedure in Hospital and Out-of-Hospital Premises Clinics: A One Year Retrospective Review of 2739 Cases During COVID19 Pandemic

**DOI:** 10.1177/22925503251334392

**Published:** 2025-04-30

**Authors:** Sophia Pei, Daniel Olteanu, Daud Manzar, Rhea Thomas, Nasimul Huq

**Affiliations:** 1 Michael G. DeGroote School of Medicine, 3710 McMaster University, Hamilton, ON, Canada; 2 Department of Surgery, 3710 McMaster University, Hamilton, ON, Canada; 3 Niagara Health - Niagara Falls Site, Niagara Falls, ON, Canada; 4 Niagara Plastic Surgery & Laser Centre, Niagara Falls, ON, Canada

**Keywords:** Complication rates, plastic surgery, pandemic, postoperative complication, adverse event, minor surgical procedure, off-hospitalpremises, chirurgie plastique, milieu extrahospitalier, complication postopératoire, événement indésirable, intervention chirurgicale mineure, pandémie, taux de complication

## Abstract

**Purpose:** This study compares complication rates for minor reconstructive procedures done under local anesthesia in a hospital setting versus out-of-hospital premises (OHP) setting during the COVID-19 pandemic. If it could be shown that minor plastic surgeries have similar, if not reduced, complication types and frequencies, this would provide a strong rationale for more procedures to be delegated to non-hospital office settings. Not only would complication rates be lower for patients, resulting in improved quality of life and health outcomes, but there would be increased efficiency for minor plastic surgery procedures, improved patient wait times, and reduced burden on hospital resources to allow for accommodation of more complex and major procedures that cannot be performed elsewhere. **Methods:** This is a retrospective medical record review of patients who underwent minor plastic surgery procedures at a community hospital and OHP settings. All procedures were performed by the same plastic surgeon. Minor plastic procedures were defined as day procedures performed with only local anesthesia. Procedures were completed with field sterility (eg, use of drapes and sterile gloves) but not room sterility. A total of 2739 charts (537 hospital charts and 2202 clinic charts) from January 2022 to December 2022, were reviewed with annotation of patient demographics, procedure type, procedure site, follow-up dates, complications, and complication type, if any. Statistical analysis involving chi-squared tests was performed on anonymized data to primarily compare complication rates between the hospital and the outpatient clinic setting, as well as secondary comparisons of subgroups such as patients with diabetes and patients using blood thinners. **Results:** There was a 3.5% complication rate for the minor procedures in the hospital compared to 1.2% in OHP setting which was a statistically significant finding. **Conclusion:** There were fewer complications for patients undergoing minor reconstructive procedures in an outpatient clinic setting versus in-hospital, indicating the potential for delegation of minor surgeries to OHP clinics and ambulatory surgery centers.

## Résumé

**Introduction :** La présente étude compare les taux de complication d'interventions reconstructives mineures effectuées sous anesthésie locale en milieu hospitalier à celles effectuées en milieu extrahospitalier (MEH) pendant la pandémie de COVID-19. Si l'on pouvait démontrer que les chirurgies plastiques mineures étaient associées à des types et des fréquences de complication semblables, sinon moins élevées, on pourrait justifier amplement l'orientation d'un plus grand nombre d'interventions en MEH. Non seulement le taux de complications serait plus faible chez les patients et améliorerait la qualité de vie et les pronostics, mais l'efficacité des interventions chirurgicales mineures s'en trouverait accrue, les temps d'attente des patients diminueraient et le fardeau sur les ressources hospitalières s'atténuerait pour laisser place aux interventions majeures plus complexes impossibles à réaliser ailleurs. **Méthodologie :** La présente analyse rétrospective de dossiers médicaux a porté sur des patients qui ont subi des interventions de chirurgie plastique mineures dans un hôpital communautaire ou un MEH. Un même chirurgien plasticien a effectué toutes les interventions. Les interventions de chirurgie plastique mineures désignaient des chirurgies d'un jour réalisées sous anesthésie locale. Le champ opératoire (p. ex., draps et gants stériles) était stérile, mais pas la salle d'opération. Les auteurs ont analysé un total de 2 739 dossiers (537 dossiers hospitaliers et 2 202 dossiers extrahospitaliers) entre janvier et décembre 2022 et ont pris note des caractéristiques des patients, du type d'intervention, du lieu d'intervention, des dates de suivi, des complications et du type de complication, le cas échéant. Ils ont procédé à l'analyse statistique à l'aide des tests du chi carré sur des données anonymisées pour comparer essentiellement les taux de complication entre le milieu hospitalier et le milieu extrahospitalier et procéder à la comparaison secondaire des sous-groupes, tels que les patients atteints de diabète et ceux qui prennent des anticoagulants. **Résultats :** Les taux de complication relatifs aux interventions mineures en milieu hospitalier s'élevaient à 3,5 % par rapport à 1,2 % en MEH, ce qui constitue un résultat statistiquement significatif. **Conclusion :** Les patients soumis à des interventions reconstructives mineures en milieu ambulatoire éprouvaient moins de complications qu'en milieu hospitalier, ce qui démontre le potentiel de diriger les interventions mineures vers des cliniques extrahospitalières et des centres chirurgicaux ambulatoires.

## Introduction

All surgical procedures carry the risk of unexpected adverse events as a result of the various factors inherent to surgery: making incisions in the skin and disrupting the dermal barrier, the use of anesthetics, the chance of iatrogenic complications such as nerve damage, and other unpredictable adverse events due to patient-specific comorbidities. The complications specific to plastic surgeries have been well established, and most commonly include surgical site infection, wound dehiscence, graft death, and arterial or nerve damage.^1^

While acknowledging that complication rates will differ based on surgery type and surgery site, the median overall incidence of in-hospital adverse events was 9.2%, with a median percentage of preventability of 43.5%.^2^ This may be due to a variety of hospital setting characteristics such as increased complexity of care, increased volume of patients, hospital-acquired infections (HAIs), and more.^3^ Meanwhile, outpatient centers’ efficacy and related frequency of adverse events are estimated to be similar, if not better, when controlling for procedure type and patient risk factors.^4^ Many procedures done in the hospital, such as carpal tunnel and trigger finger release, mucous and ganglion cyst excision, fasciectomies, skin excisions, and flap or skin graft reconstructions, can also be done with similar patient outcomes in an ambulatory surgical center. We hypothesized little difference in adverse events in these procedures conducted in hospitals versus freestanding out-of-hospital premises (OHP) clinics.

With the onset of the COVID-19 pandemic, the American College of Surgeons (ACS) laid guidelines for postponing elective and non-urgent surgeons to redirect care and resources.^5^ The backlog of surgical procedures had long-lasting effects into the years after. Between March and June 2020 was when the largest drop in surgeries was seen, however, during subsequent pandemic waves in May 2021 and January 2022, significant decreases in surgeries were also observed.^5^ Hospital overflow particularly affected wait times for plastic surgery procedures.^6^ It has been well-established that longer wait times for surgical interventions are associated with increased risks^7^ and decreased patient quality of life. Moreover, the significant cost of in-hospital post-surgical complications both to the healthcare system (eg, costs of extended care and resources) and patients themselves (eg, inability to work), represents a strong rationale for reducing post-surgical complication rates.^8^ In addition to the postponement of surgical procedures, other factors such as lack of personal protective equipment, avoidance of healthcare settings due to fear of contracting infections, staffing shortages, and healthcare worker burnout may also have contributed to the decreased number of surgical procedures performed.^5,9^ What this means for the current healthcare system is a requirement to exceed the original rates of procedures being performed.

Utilizing OHP clinics offers a solution during times when hospitals are overburdened. These clinics also can offer more patient privacy, convenience, and more consistent care from staff.^10,11^ Hence it is understandable that there has been a push for more procedures to take place in Integrated Ambulatory Centers in Ontario.^12^ If it can be shown that procedures done in these OHP settings have the same or lower rate of complications as those done in hospitals, more minor procedures can be delegated to these OHP clinics.

We have been unable to find other studies to compare the complication rates of plastic surgery minor procedures performed in ambulatory surgical centers and hospitals, controlling for the plastic surgeon. In this study, various plastic surgery minor procedures conducted in a community hospital and OHP were reviewed, and adverse event type and frequency were noted. Practical implementation of this evidence would increase efficiency for minor plastic surgery procedures, improve patient wait times and quality of life and health outcomes, and liberate hospital resources for other more complex or major procedures that could not be conducted in outpatient settings.

## Methods

A retrospective medical record review of 2739 cases undergoing minor plastic surgeries, defined as day procedures without the use of general anesthesia, was conducted.

All minor procedure cases at OHP and hospital site from January 2022 to December 2022, performed by the same surgeon, were included in the study and reviewed by researchers. Minor procedure cases were defined as procedures that can be performed with only local anesthesia, without sedation or inhalational, regional, or general anesthesia in field sterility. Data about patient age, sex, procedure type, procedure site, follow-up, complication, complication category, diabetes status, blood thinner status, and allergy status was annotated.

The procedure sites, such as finger or cheek, were further organized into broad regions such as hand/wrist or head/neck. The complication types were grouped by intervention necessary, with minor complications being those requiring reassurance alone, intermediate complications being those requiring intervention (ie, repeat procedure or prescription for antibiotics), and severe requiring hospitalization or leading to patient death. No data or outlier was removed, other than for repeat patients.

IBM SPSS version 29 was used to calculate the primary outcome of the frequency of complications across hospital and OHP cases. Analysis of associations between complication rate and contributing factors such as diabetes, use of blood thinners, and procedure site, were also completed. Given the categorical nature of most variables, Pearson Chi-square was used for exploring the statistical significance of associations. For age, as the only continuous variable, means were compared by complications in hospital and OHP, and the Student T test was used for exploring statistical significance.

## Results

In this study, 2739 minor surgical cases were reviewed during the calendar year of January 2022 to December 2022, of which 536 were hospital cases and 2202 were OHP cases. The patient demographic consisted of 54% (1486 of 2739) male and 46% (1253 of 2739) female patients; however, the percentage of males in hospital cases was even higher at 64% (344 of 537) compared to 52% (1142 of 2202) in OHP cases. The mean age for all patients was 67 years; however, patients at the hospital were older at 74 years compared to 65 years in OHP.

The most common surgical sites by region were head/neck (OHP: 49%, n = 1080; hospital 37.4%, n = 201) and hand/wrist (C 32.9%, n = 725; H 19.9%, n = 107). The 45 cases of complication between hospital and OHP settings occurred in other regions as well including the foot, leg, and torso.

**Figure 1.**
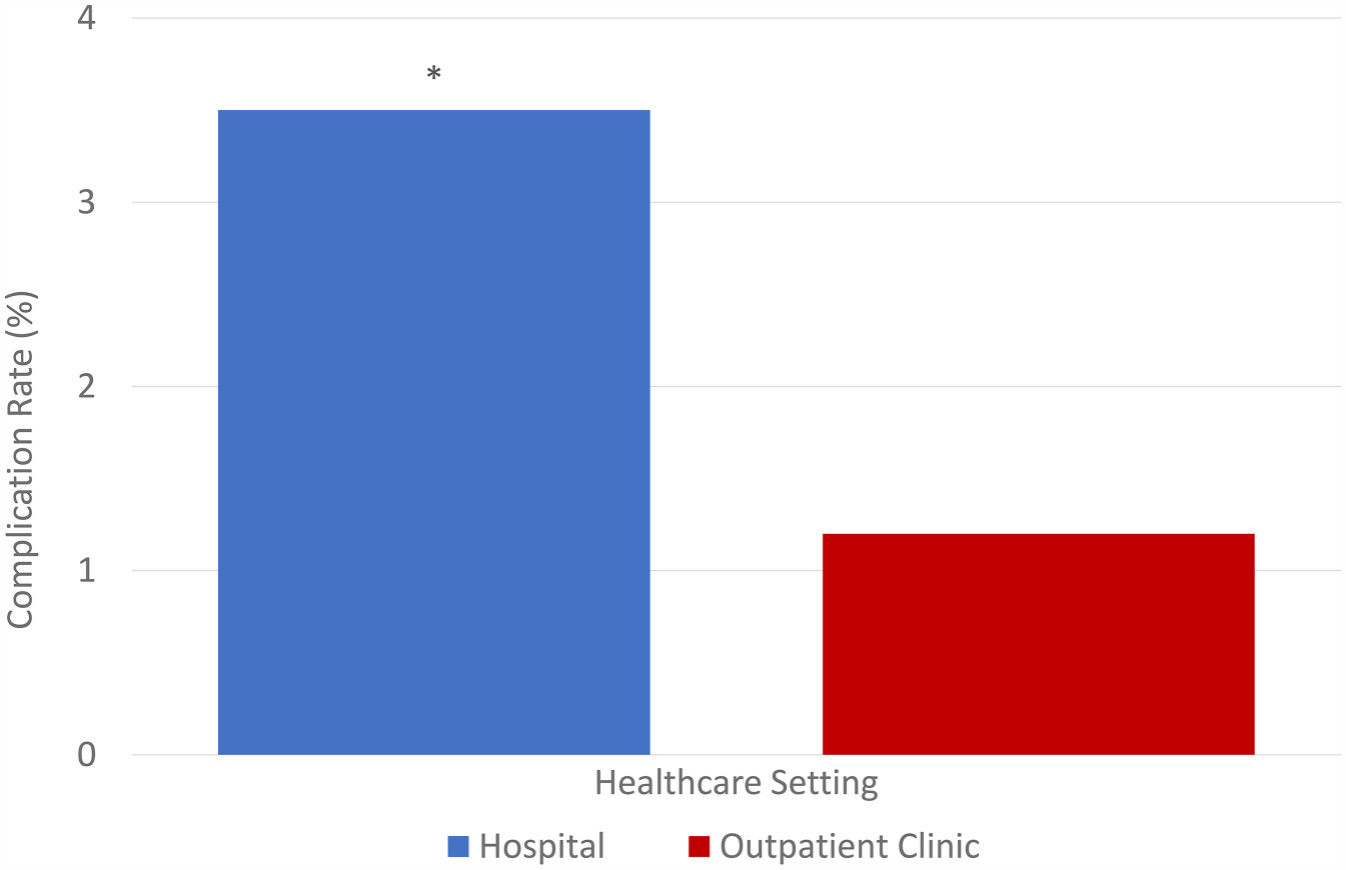
Complication rates (%) in hospital settings versus an outpatient clinic.

The primary outcome evaluated in this study was the complication rate of minor surgical procedures, dependent on whether the procedure was performed in the hospital versus in an outpatient OHP setting. The overall complication rate was 1.6% (45 of 2739). Specifically, complications in hospitals were almost triple that of OHP at 3.5% (26 of 45) compared to 1.2% (19 of 45), respectively, with Chi-square *P* = .000 as depicted in Figure 1.

**Figure 2.**
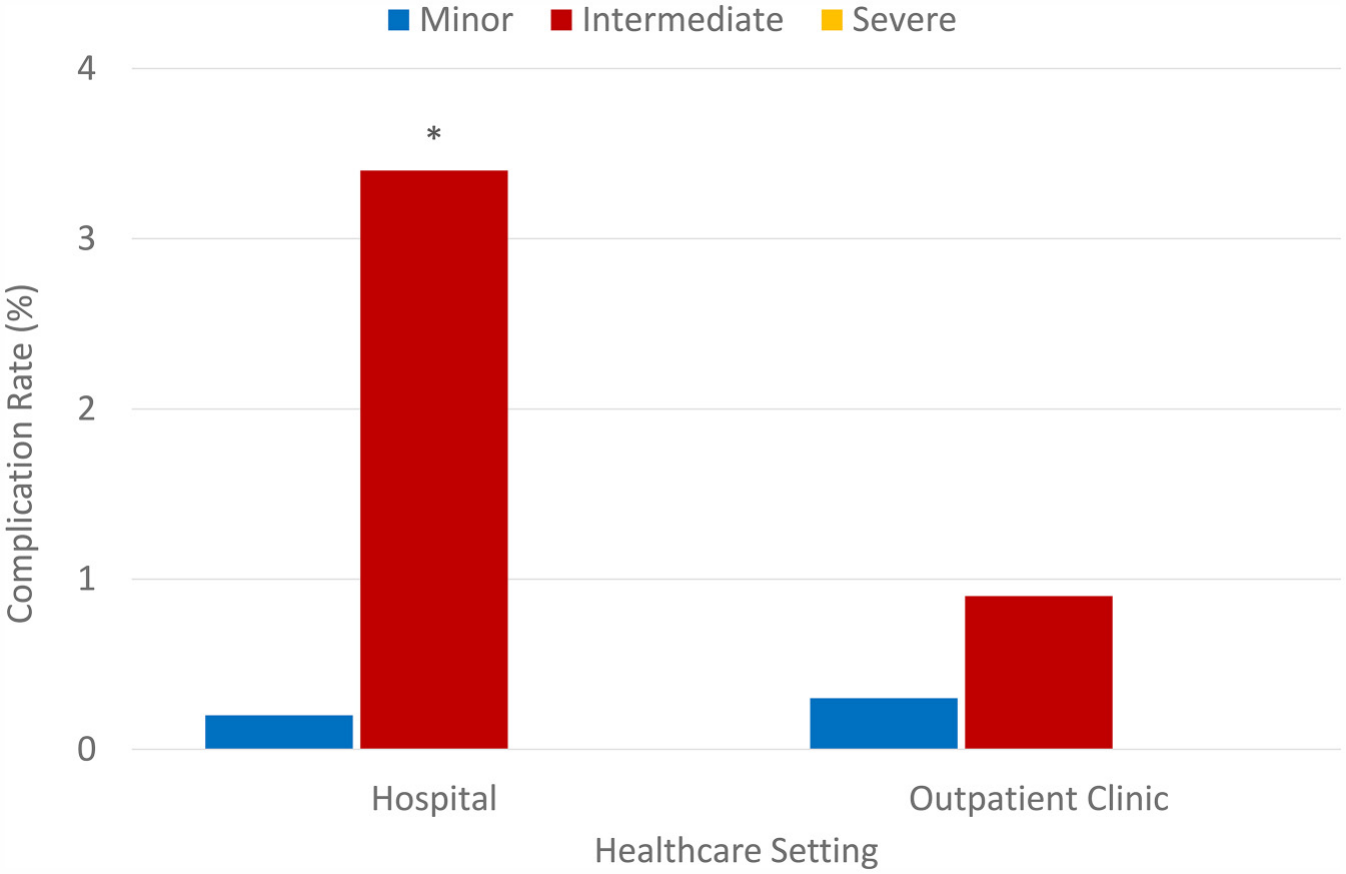
Complication rates (%), by severity, in hospital settings versus an outpatient clinic.

Out of all hospital complications, 1 was a minor while 18 were intermediate complications. Out of all OHP complications, 6 were minor while 20 were intermediate complications. These values signify that in terms of severity of complication, the hospital had triple intermediate complications than OHP (3.4% vs 0.9%) with a Chi-square *P* = .000, while proportions of minor complication were only slightly higher in OHP at 0.3% compared to 0.2% among hospital cases as shown in Figure 2. These results provide compelling evidence that hospital procedures have a significantly higher chance of developing intermediate complications rather than minor ones.

There were found to be no significant differences between complication rates of males versus females (F: 1.5%, M: 1.7%). The mean age for patients with complications was 70 versus 67 for those without complications. The mean age among those with complications was higher in the hospital at 79 years compared to 64 years at OHP.

Potentially contributing factors to the complication rate were also evaluated. In general, diabetes was a contributing factor to increased complication rates of (3.4%), compared to individuals without diabetes (1.4%). This finding was statistically significant with a Chi-square value of *P* < .001 as well as a Fisher's exact test value of 0.002. In general, blood thinner use was also associated with higher rates of complications, which was a statistically significant finding with a Chi-square value of *P* < .001 as well as a Fisher's exact test value of <0.001. The complication rates of patients with diabetes or who were on blood thinners, in regards to the healthcare setting, were also analyzed. Based on the findings, individuals who had comorbidities such as diabetes or who used blood thinners had relatively higher complication rates in a hospital setting versus OHP. The results and percentages are summarized in Table 1.

**Table 1.** Medical Comorbidities in Hospital and OHP Patient Population.

Healthcare Setting	Hospital	Off-Hospital Premises Clinic
	Diabetes	
# of Patients with condition	84 (out of 537, 15.64%)	272 (out of 2202, 12.35%)
Percentage of complication	9.5% (8 out of 84),	1.5%, (4 out of 272)
	Blood Thinners	
# of Patients with condition	201 (out of 537, 37.43%)	502 (out of 2202, 22.79%)
Percentage of complication	6.0%, (12 out of 201)	0.8%, (4 out of 502)

## Discussion

In this study, we initially hypothesized that minor plastic surgery procedures performed in OHP would have similar adverse event type and frequency to those performed in hospitals. Results found that patients undergoing minor reconstructive procedures in outpatient clinic settings experience statistically fewer complications than patients undergoing similar procedures in hospital settings (*P* < .001). Due to an overall low number of complication events within the sample population, further analysis among the population with complications could not be sufficiently powered. Of note, there were no severe complications found in either setting, and the majority of complications in both settings were intermediate and required intervention.

The statistical difference in complication rates in hospitals compared to outpatient clinics is greater than chance alone. This is likely due to multiple factors, including patient evaluation in the clinic before the procedure, as well as factors inherent to the hospital setting such as the volume of surgeries, hospital-acquired infections (HAIs), and longer hospitalization.^3,13^

Furthermore the higher ratio and quality of staff-to-patient care in outpatient settings results in lower rates of sepsis, infections, and more dependable care.^3^ Compared to the hospital, these clinics ensure a specialized, stable team working in a dedicated space. Literature shows that having the team familiarity that comes with working with the same team and having dedicated procedure rooms result in improved patient outcomes.^14^ Both the hospital and OHP procedures were performed by the same surgeon, however assisting staff such as surgical assistants, and anesthesia providers differed. Nursing staff generally cover multiple services when working at hospital sites. In contrast, the nursing staff employed at the ambulatory clinic specializes in plastic surgery. Extra familiarity could contribute to the speed of procedures and room turnover.

Another contributing aspect is that the two patient populations in the clinic and in the hospital were not randomly assigned and may represent different populations. Patient factors like age, BMI, and other comorbidities can influence postoperative complication rates.^11^ Patient factors such as race and socioeconomic status may also play a large role in complication rates and this variable should be further researched.^15^ Therefore, it is the plastic surgeon's responsibility to determine whether each patient is suitable for outpatient procedures and to adequately prepare their workplace for adverse events.^16^ Considering the nature of the procedures and the completed volume, it can be interpreted that the decision-making process applied by the surgeon in this study was intentional, appropriate, and worked as intended. This finding aligns with the expectation that the patients who receive minor procedures in the hospital and the outpatient clinic are not randomly selected, and may partially explain why the complication rates in this clinic were significantly reduced. Still, practice in an outpatient clinic-based setting should continue to review and implement evidence-based guidelines like those provided by Zhang et al to ensure patient safety is always a priority.^16^

Similar research comparing outcomes of procedures done in Independent Sector Healthcare Providers (ISHPs) versus public hospitals also found that patients treated in ISHPs had quicker discharge and lower readmission rates.^17^ Previous literature also supports that outpatient clinic settings have stricter hygiene, are not as crowded, have individual patient rooms, and well-trained staff to support the prevention of HAIs.^18,19^ This research provides compelling evidence for a shift in performing minor surgical procedures in outpatient clinics versus hospitals.

There may be other significant benefits to patient outcomes by adjusting the number of procedures done on an outpatient basis versus in the hospital. Wait times for surgical procedures have been high, and worsened by the COVID pandemic. A major contributor to these wait times is the implied necessity of operating rooms, hospital staff, and resources for these procedures, and the assumption that patients fare better when operated on in a hospital. Studies such as this one begin opening the discussion regarding the use of Integrated Ambulatory Centers in Ontario and the potential improvements they can provide to our existing healthcare system. OHP clinics allow surgeons to provide care to patients in a more timely fashion for procedures that can be safely performed outside the hospital, significantly reducing wait times and in turn, patient risk.^7^

### Limitations

One key limitation of the results is that the patient population undergoing minor procedures in the hospital was not the same as those in outpatient. All patients booked from the emergency department default to having their procedures in the hospital whereas most patients seen OHP come from referrals from general practitioners. This results in a difference in the severity of cases on presentation which could influence complications. Furthermore, all patients admitted to the hospital would subsequently have their procedures performed in hospital, leading to an overall higher rate of medical comorbidities in the hospital population. Finally, the clinic studied is not accessible for individuals with ambulatory restrictions due to the presence of stairs, and as such patients with physical disabilities would be booked to the hospital. These population subgroups may potentially have differing rates of comorbidities that were not assessed in this study.

Moreover, hospital factors alone do not provide a comprehensive overview of factors affecting complication rates. The results of this study show that diabetes and conditions requiring blood thinners, regardless of what setting, are associated with higher complication rates. In this review, the number of patients with diabetes between the hospital and OHP was comparable. Notably, the OHP had lower rates of complications concerning diabetes, whereas the hospital setting had a higher complication rate. As mentioned above, this may be due to the nature of the outpatient clinical setting that has more specialized surgical teams, more consistent patient care, stricter hygiene, less chance of HAIs, higher staff-to-patient ratios, and more. However, the number of patients on blood thinners was significantly higher than assumed by chance (*P* < .001), which may have contributed to the higher hospital complication rate in a confounding manner. While the effects of comorbidities on complication rate were examined in this study, the hospital and OHP patient populations were not matched in terms of percentage and severity of comorbidities.

Regrettably, the number and type of surgical sites (eg, arm, arm/hand/wrist, arm/head/neck) and procedure types (eg, excision/excision with graft/excision with rotation flap/excision with transposition flap) across the hospital and OHP were many, as well as overlapping in region or surgical technique. Comparison of complication rates therefore did not account for matched samples by procedure type or procedure site.

These limitations of unmatched sampling and classification bias made comparison by complication rate challenging and restricted analysis of confounding variables that may have affected it. However, a deeper and more structured analysis of complication rates by procedure type, procedure site, and concerning specific comorbidities, would serve as insightful future directions.

It also must be noted that this data was all collected from a single OHP and single hospital site, during the time frame of a pandemic. Considering the effects of a pandemic, a spike in declining hospital resources, risk of infection transmission, and diminished well-being of hospital staff might have also contributed to the higher hospital complication rates. Lastly, while there are potentially non-negligible differences within the healthcare systems, patient populations, and practitioner-specific factors, to enhance the external validity of the study, future research directions should aim to include a larger and more diverse sample size, expanding to other centers and practitioners.

### Future Directions

This retrospective chart review focused narrowly on the complication rate of minor plastic surgical procedures in hospitals versus in OHP. To further support the rationale for delegation of these procedures to OHP, evidence for benefits in regards to time, finances, and environmental impacts, as well as methods of less invasive and less taxing preparation and procedures should be pursued. Deeper inquiry into underlying reasons for, and factors affecting the complication rates in both healthcare settings should also be examined and addressed through policy and practice.

Future projects can work on quantifying the cost reduction and environmental impact of procedures in an OHP rather than the hospital. As discussed in a review by Ma et al, 20% to 70% of hospital waste has been traced back to the operating room and the shift to performing cases in an ambulatory setting may be a valuable method in reducing carbon emissions generated by the healthcare system.^20^

Lastly, more research into how complication rate is affected by surgical region and procedure type, while also matching the sample populations for comorbidities would also contribute to improving the validity of these results and facilitating the allocation of minor surgical procedures to hospitals versus OHP.

## Conclusion

This study assessed the complication rates of minor reconstructive procedures done under local anesthesia in a hospital versus OHP. Our results indicate significantly fewer complications for these procedures when performed in an OHP than in a hospital. Studies such as this can establish the safety and rationale of delegation of surgeries to such centers, opening the door to further research on the health, economic, and social improvements of this transition to the Ontario healthcare system. Particularly during times of healthcare burden, these clinics can exemplify their utility by offloading hospitals and continuing to provide patients with high-quality care.

## Supplemental Material

**Figure:** Video 1.
